# Granulocyte-colony stimulating factor producing anaplastic carcinoma of the pancreas treated by distal pancreatectomy and chemotherapy: report of a case

**DOI:** 10.1186/s40792-015-0048-y

**Published:** 2015-05-30

**Authors:** Hiroaki Kitade, Hidesuke Yanagida, Masanori Yamada, Sohei Satoi, Kazuhiko Yoshioka, Nobuaki Shikata, Masanori Kon

**Affiliations:** Department of Surgery, Kansai Medical University, Takii Hospital, 10-15 Fumizono-cho, Moriguchi, Osaka 570-8507 Japan; Department of Surgery, Kansai Medical University, 2-5-1 Shin-machi, Hirakata, Osaka 573-1191 Japan; Department of Diagnostic Pathology, Kansai Medical University, Takii Hospital, 10-15 Fumizono-cho, Moriguchi, Osaka 570-8507 Japan

**Keywords:** Granulocyte-colony stimulating factor, Pancreatic cancer, Leukocytosis, Pancreatectomy, Steroid, TS-1

## Abstract

Granulocyte-colony stimulating factor (G-CSF) producing pancreatic cancers are extremely rare. These tumors have an aggressive clinical course but no established treatment. We encountered a patient with a G-CSF-induced pancreatic cancer who was treated by surgical resection, followed by steroid treatment and chemotherapy. A 68-year-old Asian male presented at a local hospital with a 3-month history of fever, loss of appetite, and 10-kg weight loss. Laboratory data showed leukocytosis and elevation of C-reactive protein. Computed tomography (CT) revealed a 50-mm mass in the tail of the pancreas, but no signs of infective foci. He was transferred to our hospital for further evaluation. Contrast-enhanced CT showed rapid growth of this tumor over 1 week, and ^18^ F-2-fluoro-2-deoxyglucose positron-emission tomography/computed tomography (FDG PET/CT) showed FDG accumulation in the tail of the pancreas (SUV max, 17.1) but at no other sites in his body. Magnetic resonance imaging showed a heterogeneous mass, similar to that observed by CT. Three weeks later, the patient underwent a distal pancreatectomy with splenectomy. The resected specimen was 154 mm in diameter, a threefold increase from the initial image. Histopathological examination identified the tumor as an anaplastic carcinoma of the pancreas. Following surgery, his leukocyte count and body temperature were reduced. He recovered well and was discharged from our hospital on postoperative day 18. Immunohistochemical expression of G-CSF in the resected specimen and elevated serum G-CSF concentration confirmed that the mass was a G-CSF producing anaplastic carcinoma of the pancreas. Subsequently, the patient experienced a high fever and loss of appetite. CT showed recurrence of cancer in the abdominal cavity, for which he was started immediately on tegafur-gimeracil-oteracil potassium combination S-1 and steroid. Unfortunately, he died on postoperative day 83. To our knowledge, this patient was the first with a G-CSF producing anaplastic carcinoma of the pancreas to be treated by surgical resection, steroid and adjuvant chemotherapy.

## Background

Since the first description of a granulocyte-colony stimulating factor (G-CSF) producing lung cancer [1], there have been many reports of such tumors. In contrast, fewer patients with G-CSF producing digestive tract tumors, especially pancreatic cancers, have been described. The prognosis of patients with G-CSF producing pancreatic cancers is very poor [2–7]. Of the patients reported in the English language literature, only three underwent surgical operations [5–7]. To date, no standardized treatment has been established. Most patients with G-CSF producing pancreatic cancers have been treated by surgical resection or chemotherapy, not both, with others only receiving palliative care. We describe a patient with a G-CSF producing pancreatic cancer who underwent surgery followed by adjuvant chemotherapy.

## Case presentation

A 68-year-old Asian male presented at a local hospital with a 3-month history of high fever, loss of appetite, and 10-kg weight loss. He had no relevant medical history. Laboratory data showed leukocytosis, with a leukocyte count of 17,500/mm^3^, and an elevated serum C-reactive protein (CRP) concentration of 13.2 mg/dl. Computed tomography (CT) showed a mass of 50 mm in diameter at the tail of the pancreas. One week later, during which his symptoms had continued, he was transferred to our hospital for further examination.

Blood chemical findings in our hospital showed a leukocyte count of 14,900/mm^3^ and a CRP concentration of 13.5 mg/dl. His serum concentrations of aspartate aminotransferase, alanine aminotransferase, lactate dehydrogenase, creatinine, and amylase were within normal levels, but his serum alkaline phosphatase, leucine aminopeptidase, and gamma-glutamyl transpeptidase concentrations were elevated to 979, 125, and 266 U/l, respectively. Serum levels of carcinoembryonic antigen and pancreas cancer-associated antigen DUPAN-2 were within normal limits, whereas his carbohydrate antigen 19–9 concentration was elevated at 118.0 U/ml. Bacteriological examination showed no signs of infection.

**Fig. 1:**
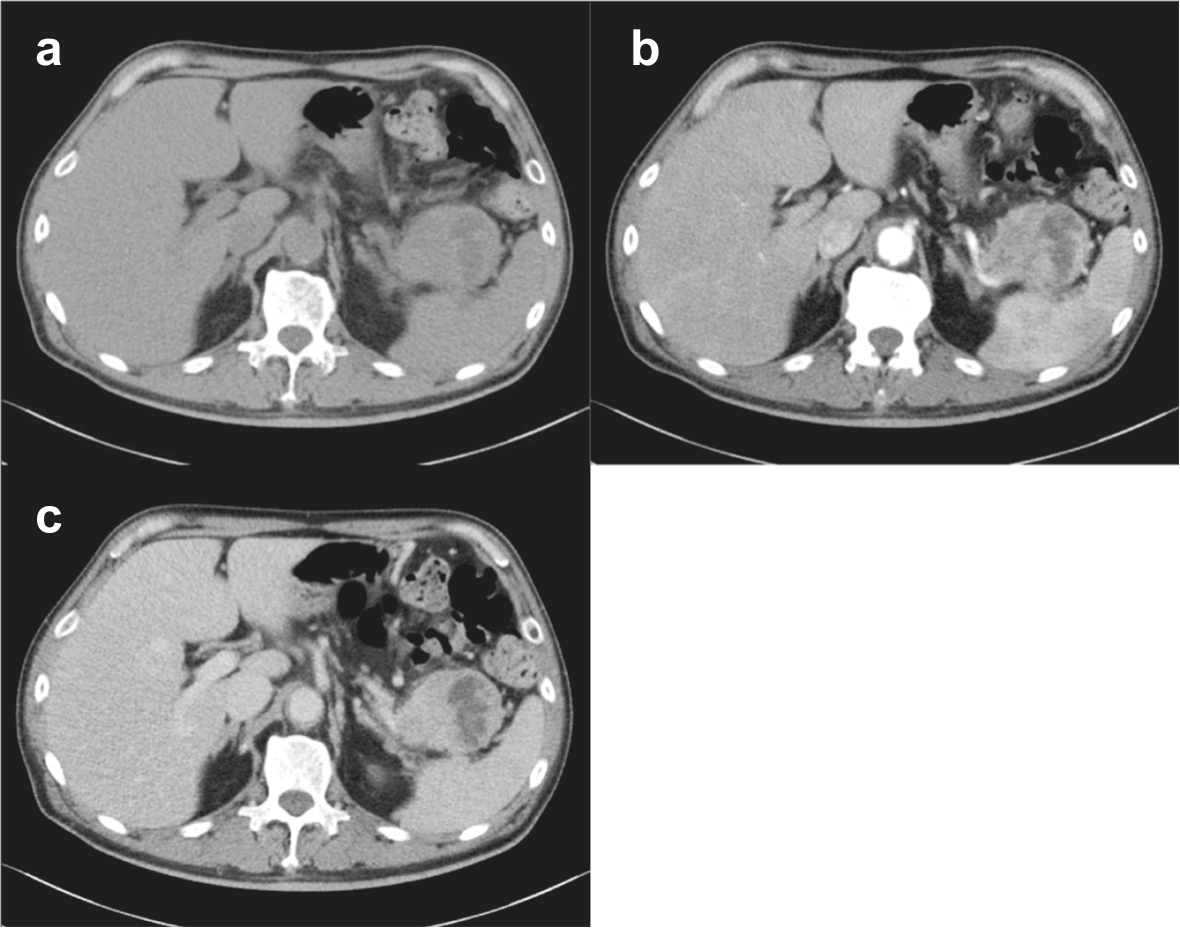
Contrast-enhanced CT on the day of first visit to our hospital, showing a heterogeneously stained tumor, 72 mm in diameter, at the tail of the pancreas. **a** plain, **b** arterial phase, **c** portal phase

**Fig. 2:**
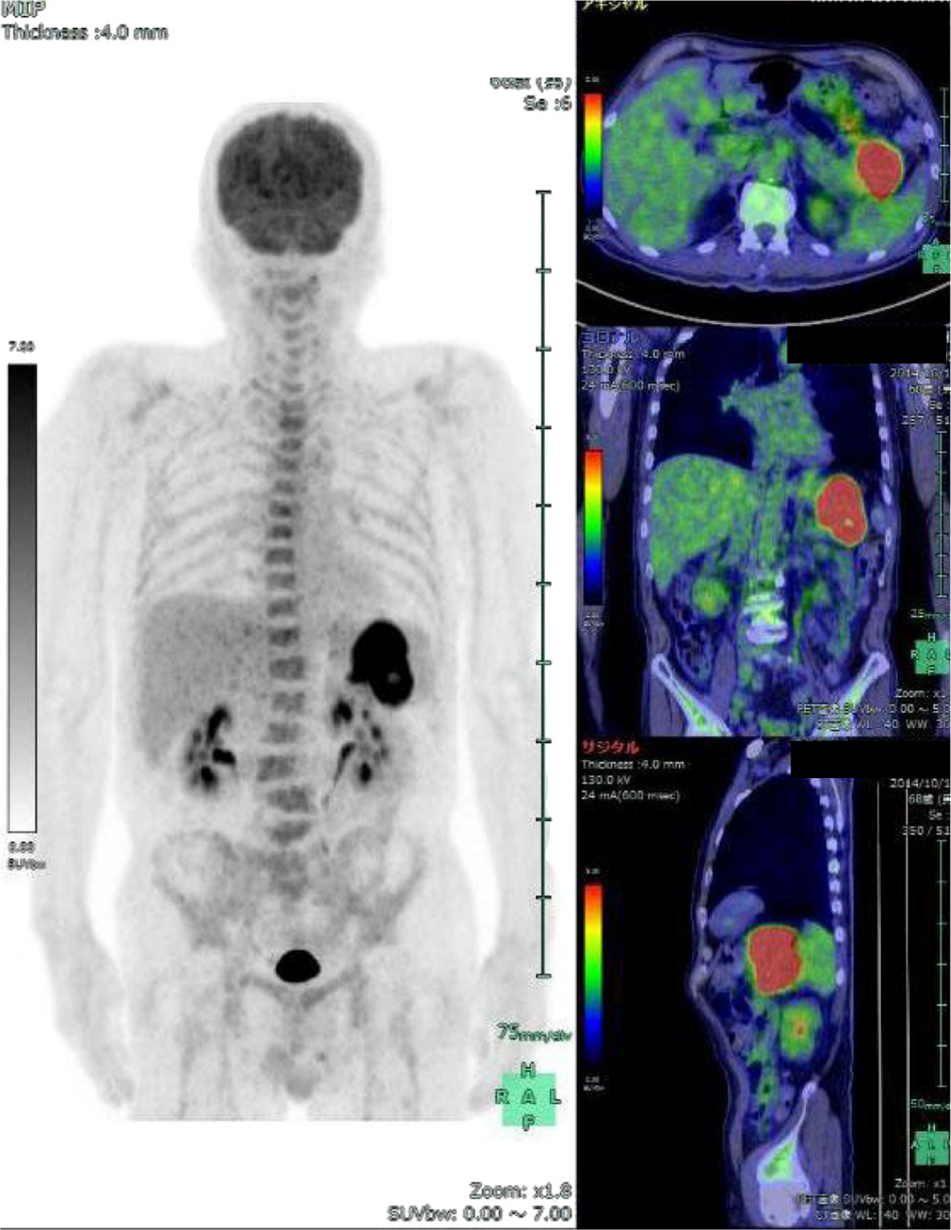
FDG PET ^18^ F-2-fluoro-2-deoxyglucose positron-emission tomography/computed tomography (FDG PET/CT) showed FDG accumulation in the tail of the pancreas (SUV, 17.1)

**Fig. 3:**
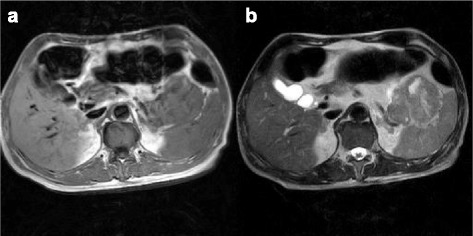
Abdominal magnetic resonance imaging (MRI) 14 days after contrast-enhanced CT. **a** T1 weighted MRI, **b** T2 weighted MRI image, showing a heterogeneous mass, 100 mm in diameter, at the tail of the pancreas

Contrast-enhanced CT showed a heterogeneously stained tumor, 72 mm in diameter, at the tail of the pancreas (Fig. 1). ^18^ F-2-fluoro-2-deoxyglucose positron-emission tomography/computed tomography (FDG PET/CT) showed FDG accumulation in the tail of the pancreas (SUV max, 17.1), but at no other sites in his body (Fig. 2). Abdominal T2-weighted magnetic resonance imaging, performed 14 days after contrast-enhanced CT, showed a heterogeneous mass at the tail of the pancreas (Fig. 3); the tumor was twice as large as in the initial CT image. Dilatation of the main pancreatic and bile ducts was not detected (Fig. 3). Gastrointestinal endoscopy showed no signs of malignancy.

**Fig. 4:**
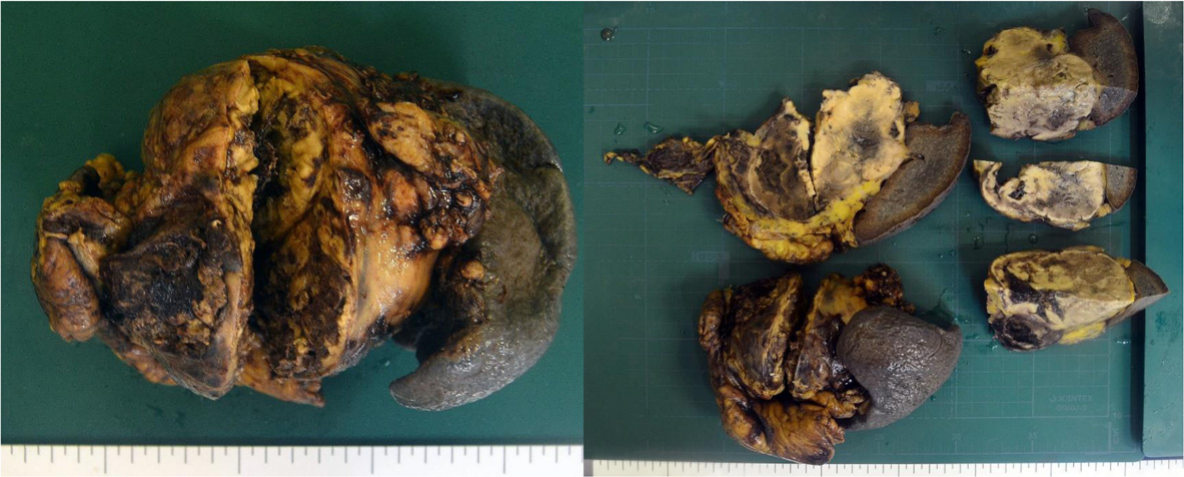
Macroscopic findings. The resected specimen was a large tumor (154 mm in diameter) at the tail of the pancreas, invading the transverse mesocolon

The rapid growth of the tumor, along with the continuous high fever, elevated leukocyte count, and elevated CRP, in the absence of infection, suggested that the mass was a G-CSF producing pancreatic cancer. The rapid growth of the tumor limited the time required to make a differential diagnosis. We therefore decided to resect the tumor, basing subsequent treatment on histopathological diagnosis. Three weeks after first presenting at our hospital, the patient underwent a distal pancreatectomy with splenectomy. Examination of frozen sections of the tumor indicated that it was an adenocarcinoma. There was no evidence of lymph node swelling or peritoneal dissemination. Intraoperative ultrasonography showed no space occupying lesion in the liver. The diameter of the resected specimen was 154 mm, three times as large as in the initial image (Fig. 4). Macroscopically curative resection was performed, despite the tumor invading the transverse mesocolon. The patient’s leukocyte count rapidly decreased from 26,800/mm^3^ on the day before the operation to 5,100/mm^3^ on postoperative day three, and his body temperature was rapidly reduced soon after the operation. He recovered well and was discharged from our hospital 2 weeks after the operation.

**Fig. 5:**
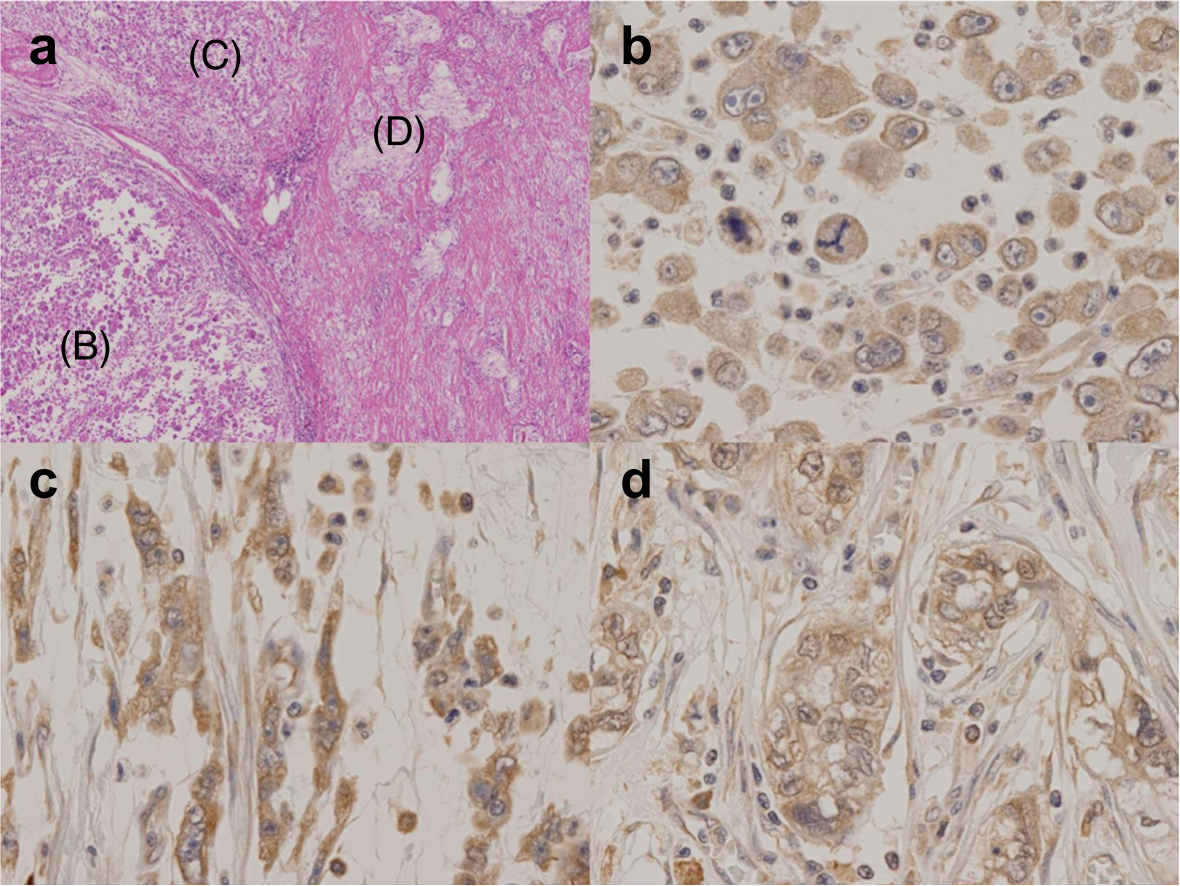
Microscopic findings. **a** The tumor was pathologically diagnosed as an anaplastic carcinoma of the pancreas, containing pleomorphic tumor cells (**b**), spindle tumor cells (**c**), and moderately differentiated ductal carcinoma (**d**) (H& E × 40). **b**-**d** Positive staining of granulocyte-colony stimulating factor (G-CSF) immunohistochemistry in the cytoplasm of formalin-fixed paraffin-embedded specimen (×400). **b** Pleomorphic tumor cell variant, composed of bizarre, multinucleated giant cells that contain abundant eosinophilic cytoplasm. The nuclei were large, hyperchromatic, and contained variable numbers and sizes of nuclei. Numerous mitoses were easily identified, including bizarre mitoses. These cells were suspended in a sea of neutrophils. **c** Spindle-cell component resembling a sarcoma, with cells arranged in fascicles, sometimes in a herringbone pattern. There was less pleomorphism than in pleomorphic tumor cells, whereas significant atypia was common. **d** Moderately differentiated ductal carcinoma

**Fig. 6:**
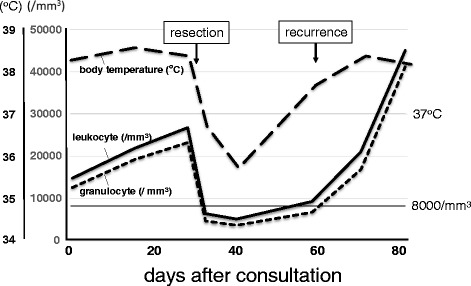
Serial leukocyte counts, granulocyte counts, and body temperature in this patient. Elevated leukocyte and granulocyte counts were reduced immediately after surgery. Body temperature was also reduced rapidly soon after the operation. These parameters were all elevated after tumor recurrence

**Fig. 7:**
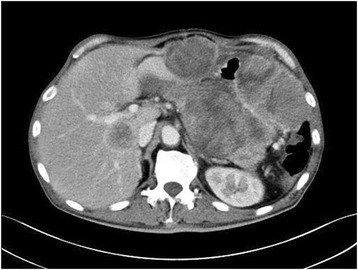
Contrast-enhanced CT at recurrence on postoperative day 36, showing peritoneal dissemination and liver metastases

Histopathological examination identified the tumor as an anaplastic carcinoma of the pancreas, composed of a ductal carcinoma component, along with bizarre giant cells and spindle-cell differentiation (Fig. 5a). Immunohistochemical examination of the resected specimen showed G-CSF expression (Fig. 5b–d), which, together with his preoperative serum G-CSF concentration of 355 pg/ml (normal range <39 pg/ml), confirmed that the tumor was a G-CSF producing pancreatic cancer. On postoperative day 48, the patient returned to our hospital with a high fever and loss of appetite. CT showed tumor recurrence. He was started immediately on tegafur-gimeracil-oteracil potassium combination S-1 (TS-1) and steroid (betamethasone) 1 mg/day. Despite administration of steroid and nonsteroidal anti-inflammatory drugs, high fever remained unaltered. Serial leukocyte counts, granulocyte counts, and body temperature are shown in Fig. 6. Two weeks after starting on TS-1, he was admitted to our hospital. Contrast-enhanced CT showed peritoneal dissemination and liver metastases (Fig. 7). His leukocyte count and serum CRP concentration increased. He was continued on steroid and TS-1 for 3 weeks, but his condition worsened, and he died on postoperative day 83.

## Conclusions

This report describes a very rare case of G-CSF producing anaplastic carcinoma of the pancreas. To date, only six G-CSF producing pancreatic cancers have been reported in the English language literature [2–7], including three anaplastic carcinomas, one adenosquamous carcinoma, and two poorly differentiated adenocarcinomas. Early suspicion of the nature of this tumor made possible its resection despite its very rapid growth. The combination of surgery, chemotherapy, and steroid treatment in this patient did not improve his outcome.

Following the first report of a G-CSF producing tumor in a patient with lung cancer [1], G-CSF producing tumors were reported in various organs [8–10]. G-CSF producing pancreatic cancers account for 6.8 % of all G-CSF producing tumors [11]. Patients with G-CSF producing tumors have elevated serum G-CSF and increased leukocyte counts, which are reversed by tumor resection. Moreover, G-CSF is expressed in the resected specimen, as shown by immunohistochemistry (IHC) and/or reverse-transcriptase polymerase chain reaction. Our patient met all of these criteria for a G-CSF producing tumor. Furthermore, leukocytosis recurred at the time of tumor recurrence. Only one other patient with a G-CSF producing pancreatic tumor who underwent surgical resection was found to have tumor G-CSF expression by IHC. The other five patients underwent either resection or IHC.

Microscopic examination of this tumor showed that it was an anaplastic carcinoma of the pancreas. Anaplastic carcinoma of the pancreas, first described in 1954 [12], can be classified into the following four types: 1) giant cell type; 2) osteoclast-like giant cell type/giant cell carcinoma of osteoclastoid type; 3) pleomorphic type; and 4) spindle-cell type [13]. The tumor in our patient was the pleomorphic type, but also contained other cell types (Fig. 5a). Bizarre giant cell and spindle-cell components could be differentiated from the ductal carcinoma component immunohistochemically, G-CSF is expressed in bizarre giant cell and spindle-cell cytoplasm, as in moderately differentiated ductal carcinoma cells (Fig. 5a–d). Only 0.25 % of all pancreatic cancers are anaplastic carcinomas [14]. The mean age of these patients has been reported to be 61.1 years, with a male-to-female ratio of 24:1 and a mean survival of 2–7 months [15–17]. Patients with G-CSF producing anaplastic carcinomas were reported to be of mean age 64 years, with a male-to-female ratio of 3:4, and a mean survival time of 85 days [18]. IHC in our patient showed G-CSF expression by various types of cells. *KRAS* mutant allele-specific imbalance has been reported to correlate with progression to undifferentiated carcinoma of the pancreas [19], and expression of granulocyte/macrophage colony stimulating factor (GM-CSF) to correlate with *KRAS* mutations in pancreatic tumors [20]. G-CSF producing tumors may be affected by GM-CSF.

Growth of this tumor is very rapid, precluding surgical resection in many patients. The tumor in our patient was resected because we suspected that it was an early stage cancer. At resection, the tumor was three times as large as in the initial image taken 1 month earlier. Had we waited the normal 1–2 months, the tumor could not have been resected.

The prognosis of patients with G-CSF producing pancreatic cancer is poor, the longest overall survival time being 270 days after initial consultation [21]. The mean overall survival from detection to death has been reported to be 81.2 days [3]. Treatments reported for this type of cancer include surgical resection [5, 7] and chemotherapy [22], with some patients eligible only for palliative care. Our patient underwent surgical resection followed by treatment with tegafur-gimeracil-oteracil potassium combination S-1 (TS-1) and steroid. Although TS-1 treatment was reported to reduce FDG accumulation, that patient survived only 88 days [22]. TS-1 plus gemcitabine chemotherapy and steroid reduced the leukocyte count in one patient [23]. Our patient was administered steroid (betamethasone) to reduce fever, enhance appetite, and suppress G-CSF and IL-6 expression. Although this patient experienced a transient increase in appetite, his high fever and tumor growth continued, and leukocytosis did not improve, suggesting that surgical resection followed by TS-1 and steroid was ineffective.

In summary, surgical resection, followed by steroid plus standard adjuvant chemotherapy, was not effective in this patient with a G-CSF producing anaplastic carcinoma of the pancreas. New treatment strategies are needed for these patients.

## Consent

Written informed consent was obtained from the patient for publication of this case report and accompanying images. A copy of the written consent is available for review by the Editor-in-Chief of this journal.

## Abbreviations

G-CSF: granulocyte-colony stimulating factor
CT: computed tomography
FDG: ^18^ F-2-fluoro-2-deoxyglucose
PET: positron-emission tomography
TS-1: tegafur-gimeracil-oteracil potassium combination S-1
CRP: C-reactive protein
IHC: immunohistochemistry
